# LILACS, mission and abstracts

**DOI:** 10.1590/S1980-57642009DN30200001

**Published:** 2009

**Authors:** Ricardo Nitrini

**Affiliations:** Editor-in-Chief

**Dementia & Neuropsychologia** has recently been indexed in the Literature on the Health Sciences in Latin America and the Caribbean (LILACS) database, making information contained in the papers we publish more visible to the regional scientific community. Our goal is to be indexed in other international databases over the forthcoming months. Together with the free full text availability provided by our site *demneuropsychol.com.br*, we are confident we are going in the right direction toward making our journal a better vehicle for publication of manuscripts from Latin America and elsewhere.

**Dementia & Neuropsychologia** carries on its mission of publishing research on aspects of dementia and neuropsychology that are pertinent to developing countries, particularly issues relevant to populations with low or heterogeneous educational background. In this issue, we publish two papers reporting the influence of educational level on performance in two well-known tests: the Mini-Mental State Examination and the Mini-Cog.

Since its launch, **Dementia & Neuropsychologia** has published abstracts of several symposia held in Brazil, but we aim to publish abstracts of events from other countries in Latin American and abroad. Carrying these presentations is a significant part of our mission, because abstracts of meeting presentations are hard to find when not published as special supplements or part of a journal.

In this issue, we are publishing the abstracts of the IPA 2009 International Meeting held in Rio de Janeiro on 04-07 May, and sponsored by the International Psychogeriatric Association in cooperation with the Brazilian Association of Geriatric Neuropsychiatry (ABNPG). Hosting this International Meeting in Brazil is a significant achievement for the ABNPG in its effort toward raising the quality of scientific research in the field and providing Brazilian and Latin American researchers with an opportunity to participate as speakers in international congresses and symposia.

Publishing the abstracts of the IPA 2009 International Meeting represents a significant milestone, enabling platform and poster presentations to reach a broader scientific community. We regard abstracts as the forerunners of potential full manuscripts for future publication in **Dementia & Neuropsychologia**.

**Ricardo Nitrini** Editor-in-Chief

